# Effect of intermittent isometric handgrip exercise protocol with short exercise duration on cognitive performance

**DOI:** 10.1186/s12576-021-00796-z

**Published:** 2021-04-06

**Authors:** Shotaro Saito, Takuro Washio, Hironori Watanabe, Soichi Ando, Shigehiko Ogoh

**Affiliations:** 1 Department of Biomedical Engineering, Toyo University, 2100 Kujirai, Kawagoe-shi, Saitama, 350-8585 Japan; 2 Research Fellow of Japan Society for the Promotion of Science, Tokyo, Japan; 3 Graduate School of Informatics and Engineering, The University of Electro-Communications, Tokyo, Japan; 4 Neurovascular Research Laboratory, University of South Wales, Pontypridd, UK

**Keywords:** Static exercise, Go/No-Go task, Memory recognition task, Exercise pressor reflex, Executive function

## Abstract

The handgrip exercise, a small muscle exercise, is useful for exercise therapy, particularly in the elderly and bedridden patients. The isometric handgrip (IHG) exercise has been utilized in training programs to reduce resting blood pressure; however, the acute effects of the IHG exercise on cognitive performance are not fully understood. The present study aimed to investigate the effect of an intermittent IHG exercise protocol with short exercise duration, which minimizes the arterial blood pressure response to exercise, on cognitive performance. Twenty-two young healthy subjects performed the intermittent IHG exercise protocol, which consisted of 30-s IHG and 45-s recovery × 16 trials; the exercise intensity of the IHG exercise was 30% of the maximal voluntary contraction. Cognitive performance was evaluated before and after the exercise with the Go/No-Go and memory recognition tasks. Specifically, the reaction time (RT) and performance accuracy were measured. The intermittent IHG exercise protocol did not change the RT or performance accuracy of either the Go/No-Go task (*P* = 0.222 and *P* = 0.260, respectively) or the memory recognition task (*P* = 0.427 and *P* = 0.245, respectively). These findings suggest that the intermittent IHG exercise protocol with short exercise duration may not provide enough stimulation to improve cognitive performance despite being useful as a safe exercise therapy in the elderly and in patients with cardiovascular disease.

## Background

Cognitive function is an essential component of most daily activities [1, 2], but it is changeable and determined by many physiological factors. For example, aging and cardiovascular disease have been reported to attenuate cognitive function [3, 4]. Therefore, by one method or another, cognitive dysfunction must be prevented to maintain quality of life for the elderly and any other patients [5]. Previous studies have reported that both dynamic and static (isometric) exercise training improved cognitive performance and cardiovascular function [6–9]. However, in general, dynamic exercise rather than isometric exercise is recommended as exercise therapy in the elderly or any patients who have a higher blood pressure response to exercise [10–15] because the dynamic exercise-induced increase in arterial blood pressure (ABP) is lower than that induced by isometric exercises [16].

Importantly, it is difficult in some cases for the elderly and patients who have limitations moving large muscle groups to perform dynamic whole-body exercises, e.g., cycling, walking, etc. On the other hand, it has been reported that exercise training using the isometric handgrip (IHG) exercise protocol (2-min IHG at 30% maximal voluntary contraction (MVC) and 3-min recovery × 4 trials) reduces resting blood pressure and improves arterial stiffness as well as endothelial function [17–24]. Moreover, our recent study demonstrated that the acute IHG exercise protocol improved cognitive performance [25]. These findings indicate that the IHG exercise may be a better exercise mode for cardiovascular and cognitive therapies, particularly in the elderly and bedridden patients, compared with dynamic whole-body exercise because the IHG exercise is a useful small muscle exercise that can be easily performed in various conditions, such as in a supine position in a hospital bed.

One important problem using an isometric small muscle exercise for the elderly and patients with cardiovascular disease is that their exercise pressor response is larger compared with healthy young adults [10, 26, 27]. Exercise with a large blood pressure response is often contraindicated in patients with cardiovascular disease because an unusually large increase in blood pressure during exercise may increase the risk of cardiovascular events. In addition, the IHG exercise may augment the blood pressure response more than dynamic whole-body exercise [16].

It is noteworthy that a previous study [28] indicated that in the IHG exercise protocol, the ABP response can be modified by maintaining the total exercise volume while decreasing the contraction time and increasing the frequency of IHG contractions. Indeed, our pilot study [29] confirmed that the intermittent IHG exercise protocol with short duration maintaining the same total exercise volume as the traditional IHG exercise protocol resulted in a 26% lower pressor response to exercise (mean ABP; 99 mmHg vs. 124 mmHg, *P* = 0.007, n = 7) compared with that of the IHG exercise protocol that reduces resting ABP as reported previously [19, 20]. More recently, our study [25] demonstrated that the acute traditional IHG exercise protocol improved cognitive performance. Moreover, previous studies [30–32] using dynamic exercise with whole body or large muscle groups suggest that the same total exercise volume would have an equally positive effect on cognitive performance regardless of different exercise duration and frequency. Therefore, we hypothesized that the acute intermittent IHG exercise protocol with short exercise duration that maintains the total exercise volume as the traditional IHG protocol [25] improves cognitive performance with a lower blood pressure response.

In the present study, we investigated the acute effect of the intermittent IHG exercise protocol with short exercise duration (30-s IHG at 30% MVC and 45-s recovery × 16 trials) on cognitive performance. The findings of the present study are expected to provide important information as to whether the intermittent IHG exercise with short exercise duration is a useful exercise mode for preventing cognitive dysfunction, particularly in the elderly and any patients who have a high exercise pressor reflex and limitations with moving large muscles.

## Methods

### Ethical approval

This experimental protocol was approved by the Institutional Review Board at Toyo University (Approval Number: TU2019-039), and each subject provided written informed consent prior to participation in accordance with the principles of the Declaration of Helsinki.

### Subjects

Twenty-two healthy volunteers participated in this study (18 men and 4 women; mean age, 22.0 ± 1.0 years; height, 169.6 ± 7.9 cm; weight, 60.9 ± 10.4 kg). None of the subjects had cerebrovascular or cardiovascular disease; all subjects were not taking any medications and were non-smokers. Each subject abstained from caffeine for 12 h and strenuous exercise and alcohol for 12 h. The experiment was performed at least 4 h after a light meal.

### Experimental procedure

On the same day, before the experiment, all subjects practiced the intermittent IHG exercise protocol and completed at least one practice block of each task (Go/No-Go tasks; 30 trials and memory recognition; 10 words) to minimize learning effects. The intermittent IHG exercise protocol with short exercise duration (EX) and control condition (CON) were performed on different days randomly to counterbalance across the subjects. On the day of the CON, each subject was asked to read a Japanese scientific magazine for the same experimental period as the EX (20 min) instead of performing the EX. On the day of the EX, each subject performed three repetitions of their MVC of the IHG exercise on the left hand (non-dominant hand) to determine the exercise intensity for the experiment. After a sufficient resting time (more than 30 min), each subject performed the EX, which consisted of sixteen 30-s isometric contractions at 30% of MVC separated by 45-s rest between IHG exercise trials with the non-dominant hand. In both conditions, before and after the EX and CON, the subjects performed the cognitive tasks. All studies were performed experiments on the same time (8AM–13PM) of day for both conditions at a constant temperature (24℃). In our pilot study [29], we confirmed that the response of the mean arterial pressure to the EX (intermittent IHG exercise protocol) was lower (99 ± 11 mmHg vs. 124 ± 8 mmHg, *P* = 0.007, n = 7) than that of the traditional IHG exercise protocol (four 2-min isometric contractions at 30% of MVC separated by 3 min of rest) used previous study [33].

### Measurements

#### Cognitive performance (Go/No-Go tasks, memory recognition task)

Cognitive performance in this study was measured using the Go/No-Go tasks [34] and the memory recognition task [35], which require executive function and memory recognition, respectively. To evaluate cognitive performance, the mean reaction time (RT) and performance accuracy of the Go/No-Go task and memory recognition task were calculated.

*Go/No-Go task:* each trial started with a blank screen for 2.5 s, followed by a reparatory stimulus (green square) presentation at the center of the computer screen for 1 s. Then, one square of several possible colors (red, blue, yellow, or purple) was presented at the center of the computer display for 1 s. The red and blue squares represented the Go signal, and the yellow and purple squares represented the No-Go signal. If the Go signal was displayed, the subjects were asked to press the left button of the computer mouse with their right index finger as quickly as possible. If the No-Go signal was displayed, the participants were asked to refrain from responding. The Go/No-Go task consisted of 60 trials with equal probability (30 Go trials and 30 No-Go trials).

*Memory recognition task:* first, 30 Japanese words were presented at a rate of 1 word per 1 s for memorization. Approximately 5 min after the Go/No-Go task, the subjects performed the memory recognition portion of the test. To assess memory recognition performance, 60 words (30 distracters) were presented every 2 s, and at each word presentation, the subjects answered as quickly as possible whether that word was presented during the memorization period.

### Cardiovascular measures

Heart rate (HR) was measured using a lead II electrocardiogram (bedside monitor, BMS-3400; Nihon Kohden, Tokyo, Japan). ABP was monitored continuously using a finger photoplethysmography (Finapres Medical Systems, Amsterdam, the Netherlands). Systolic blood pressure (SBP), diastolic blood pressure (DBP), and mean arterial pressure (MAP) were obtained from the ABP waveform. The HR, SBP, DBP, and MAP data were averaged using 30-s data points at the resting baseline and end of the intervention (last set). To identify the change in these parameters during EX, 30 s averages were calculated during the 4th, 8th and 12th sets of EX. The psychological arousal level was evaluated using the felt arousal scale (FAS; 1: low arousal to 6: high arousal) immediately after the cognitive tasks [36].

### Statistical analysis

The values are expressed as the mean ± SD. Changes in HR, SBP, DBP, and MAP during EX were assessed with a one-way repeated-measures analysis of variance (ANOVA). Regarding the data of CON, changes in these values were assessed using a paired t-test because of two data points (the baseline and end of experimental period). Absolute changes in the RT and performance accuracy of the cognitive task and the arousal level during the Go/No-Go and memory tasks were assessed by a two-way (time: pre- and post-test × condition: CON and EX) repeated-measures ANOVA. The effect size were calculated as *eta-squared (η*^*2*^) for all ANOVA outcome or *Hedges’ g*_*av*_ for t-test using the spreadsheet provided by Lakens [37]. The ANOVAs were followed by the Bonferroni's multiple post hoc test. A *P*-value < 0.05 was considered significant.

## Results

**Fig. 1:**
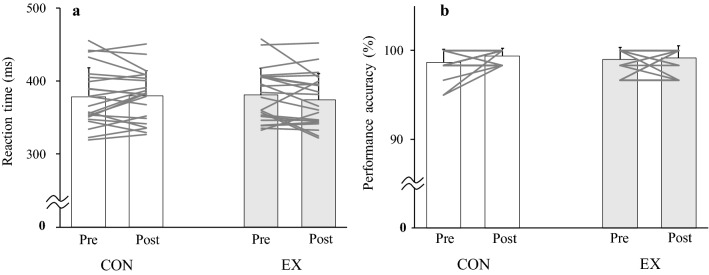
Reaction time (**a**) and performance accuracy (**b**) of the Go/No-Go task in the CON (control condition) and EX (intermittent IHG exercise protocol with short duration) (*n* = 22)

**Fig. 2:**
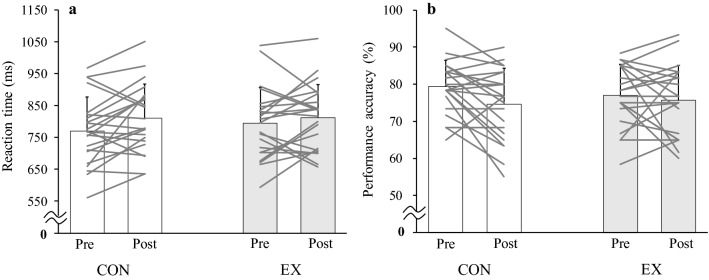
Reaction time (**a**) and performance accuracy (**b**) of memory recognition task in the CON (control condition) and EX (intermittent IHG exercise protocol with short duration) (*n* = 22)

The cardiovascular responses to the EX and CON are shown in Table 1. In the CON, the cardiovascular responses did not change from the baseline value (*t*_21_ > 0.359, *P* > 0.271, *Hedges’ g*_*av*_ > 0.052). During the EX, HR did not increase (*F*_1,21_ = 1.246, *P* = 0.298, *η*^*2*^ = 0.010). Conversely, SBP, DBP, and MAP increased (*F*_1,21_ > 21.214, *P* < 0.001, *η*^*2*^ > 0.182). The psychological response during the cognitive tasks is shown in Table 2. The FAS remained unchanged during the cognitive tasks in the CON, but increased in the EX (*F*_1,21_ = 4.915, *P* = 0.038, *η*^*2*^ = 0.060); post hoc comparisons using the Bonferroni test indicated that the increase in the FAS during cognitive tasks was observed after the EX (*P* = 0.013). However, the EX did not change the RT or performance accuracy in the Go/No-Go task (*F*_1,21_ = 1.581, *P* = 0.222, *η*^*2*^ = 0.020 and *F*_1,21_ = 1.340, *P* = 0.260, *η*^*2*^ = 0.027, respectively, Fig. 1) or in the memory recognition task (*F*_1,21_ = 0.657, *P* = 0.427, *η*^*2*^ = 0.012 and *F*_1,21_ = 1.428, *P* = 0.245, *η*^*2*^ = 0.020, respectively, Fig. 2) despite an exercise-induced increase in the FAS.

**Table 1:** Cardiovascular measurement at resting baseline, end of the intervention (last set) and during intermittent isometric handgrip exercise protocol with short duration

	Baseline	4th set	8th set	12th set	Last set	*P* values
HR, beats/min						
CON	67 ± 9	-	-	-	66 ± 9	*P* = 0.723
EX	67 ± 8	67 ± 6	68 ± 8	68 ± 7	69 ± 7	*P* = 0.298
SBP, mmHg						
CON	116 ± 12	-	-	-	118 ± 11	*P* = 0.271
EX	116 ± 7	127 ± 11*	127 ± 10*	126 ± 12*	127 ± 11*	*P* < 0.001
DBP, mmHg						
CON	69 ± 7	-	-	-	71 ± 8	*P* = 0.306
EX	67 ± 5	76 ± 8*	76 ± 7*	76 ± 8*	76 ± 9*	*P* < 0.001
MAP, mmHg						
CON	88 ± 9	-	-	-	89 ± 9	*P* = 0.290
EX	86 ± 5	96 ± 9*	96 ± 9*	96 ± 10*	96 ± 10*	*P* < 0.001

Values are mean ± SD (*n* = 22)

*HR* heart rate, *SBP* systolic blood pressure, *DBP* diastolic blood pressure, *MAP* mean arterial pressure, *CON* control condition, *EX* intermittent IHG exercise protocol with short duration. ^*^P < 0.05 vs. Baseline

**Table 2:** The psychological arousal level during the cognitive tasks

	CON		EX		*P* value (interaction)
FAS	Pre	Post	Pre	Post	*P* = 0.038
	3.0 ± 1.0	3.0 ± 0.7	2.9 ± 0.9	3.4 ± 0.9^*†^	

Values are mean ± SD (*n* = 22)

*FAS* felt arousal scale, *CON* control condition, *EX* intermittent IHG exercise protocol with short duration. ^*^P < 0.05 vs. Pre, ^†^*P* < 0.05 vs. CON

## Discussion

The intermittent IHG exercise protocol with short exercise duration, which minimizes the blood pressure response, did not improve cognitive performance despite causing an exercise-induced increase in the psychological arousal level. This intermittent IHG protocol is useful as a safe and effective exercise mode in the elderly and in patients with cardiovascular diseases who have a higher risk of cardiovascular events; however, this exercise stimulation is not sufficient for improving cognitive performance. To improve the clinical applicability of these findings, we must further investigate and identify an adequate mode of small muscle exercise that can be applied to safely improve cognitive performance for the elderly and patients with cardiovascular disease.

Some epidemiological longitudinal studies [38, 39] have reported that both dynamic and isometric exercise training (chronic exercise influence) improved cognitive performance, and thus confirm the validity of the findings presented in epidemiological cross-sectional study [40]. The mechanism of these exercise training-improved cognitive function remains unknown, but previous findings suggest that the physiological adaptation to exercise training, e.g., direct (brain volume) [41] and indirect (resting blood pressure, insulin resistance, physical fitness) physiological factors [38, 39, 42–44], is associated with the improvement in cognitive function.

On the other hand, investigating the effect of acute exercise (one bout of exercise) on cognitive performance can be isolated from the chronic exercise-induced effects and consequently provides important information regarding the acute influence of exercise on brain function because one bout of exercise does not alter brain volume, insulin resistance, physical fitness, etc. Under these backgrounds, many previous studies have investigated the acute effect of one bout of exercise on cognitive performance. One previous study [45] reported that dynamic exercise improves cognitive performance. The authors also manipulated cerebral blood flow, but changes in cerebral blood flow did not modify cognitive performance. Thus, the authors concluded from these results that acute exercise-stimulated neural activation rather than cerebral metabolism improved cognitive performance. Another study [46] demonstrated that cognitive performance was improved after acute high-intensity interval training, and this improvement is associated with exercise-induced lactate production. It is noteworthy that these acute exercise-induced improvements in cognitive function occur without a chronic physiological adaptation, for example, caused by exercise training.

Conversely, the acute effect of small muscle exercises on cognitive performance is poorly understood. Our recent study [25] demonstrated that the traditional IHG exercise protocol improved cognitive performance. In addition, a previous study reported that an acute dynamic exercise-induced increase in arousal level was associated with increased brain neural activity and cognitive performance [47]. However, in contrast to these previous studies and our hypothesis, the intermittent IHG exercise protocol with short exercise duration did not improve cognitive performance despite an increase in the FAS (arousal level). This finding highlights two important points in this research area. First, small muscle exercise may be different from large muscle exercise with regard to improvement in cognitive performance. One previous study [48] that reported on an acute isometric exercise using a larger muscle (leg extension–quadriceps femoris muscle), compared with IHG exercise, noted improved cognitive performance. Thus, small muscle exercises may be limited in their ability to improve cognitive performance. Indeed, the increase in the FAS during exercise in the present study was lower than that during the large muscle isometric exercise (0.5 vs 1–2) [48]. Second, we used the intermittent IHG exercise protocol with short exercise duration to prevent a large blood pressure response, and during this exercise protocol, the HR sample entropy was larger than that of the traditional IHG exercise protocol, which included longer contractions (2-min at 30% MVC IHG and 1-min recovery × 4 trials, corresponding to the same amount of exercise as the intermittent IHG exercise protocol in this study) [28]. These findings indicate that the intermittent IHG exercise protocol in the present study provided a lower sympathetic stimulus and thus autonomic stimulation may be not enough to improve cognitive performance. Importantly, our previous study found that the traditional acute IHG exercise protocol (2-min at 25% MVC IHG and 3-min recovery × 4 trials) increased blood pressure to a larger extent and improved cognitive performance [25]. Thus, the IHG exercise may be a useful exercise mode to improve cognitive performance, but we must consider an adequate exercise protocol (strength, duration, frequency and so on) using IHG to safely improve cognitive performance. To perform this rehabilitation exercise in the elderly and in patients with cardiovascular diseases, we must further investigate and establish an adequate IHG exercise protocol based on the balance between sufficient autonomic stimulation and preventing a large blood pressure response.

Potential limitations of the present study should be considered. Since we recruited only young healthy adults, the present results cannot be generalized to older or hypertensive individuals. However, given that exercise training-induced cardiovascular adaptations improve cognitive performance, older and hypertensive individuals may gain a large cognitive improvement through exercise therapy. Further investigations regarding this point in the clinical research area must be performed.

## Conclusions

In contrast to our hypothesis, the intermittent IHG exercise protocol with short exercise duration did not improve cognitive performance despite causing an exercise-induced increase in the psychological arousal level. To improve the clinical applicability of these findings, we must further investigate and establish an adequate IHG exercise protocol based on the balance between sufficient autonomic stimulation and preventing a large blood pressure response.

## Data Availability

The datasets used and analyzed during current study are available from the corresponding author on reasonable request.

## References

[1] Fiocco AJ, Yaffe K (2010). Defining successful aging: the importance of including cognitive function over time. Arch Neurol.

[2] Logsdon RG, McCurry SM, Teri L (2007). Evidence-based interventions to improve quality of life for individuals with dementia. Alzheimers Care Today.

[3] Breteler MM, Claus JJ, Grobbee DE, Hofman A (1994). Cardiovascular disease and distribution of cognitive function in elderly people: the Rotterdam Study. BMJ.

[4] Kilander L, Nyman H, Boberg M, Hansson L, Lithell H (1998). Hypertension is related to cognitive impairment: a 20-year follow-up of 999 men. Hypertension.

[5] Song D, Yu DS, Li PW, He G, Sun Q (2019). Correlates of health-related quality of life among chinese older adults with mild cognitive impairment. Clin Interv Aging.

[6] Alghadir AH, Gabr SA, Al-Momani M, Al-Momani F (2020). Moderate aerobic training modulates cytokines and cortisol profiles in older adults with cognitive abilities. Cytokine.

[7] Dempster DW, Brown JP, Fahrleitner-Pammer A, Kendler D, Rizzo S, Valter I, Wagman RB, Yin X, Yue SV, Boivin G (2018). Effects of long-term denosumab on bone histomorphometry and mineralization in women with postmenopausal osteoporosis. J Clin Endocrinol Metab.

[8] Weuve J, Kang JH, Manson JE, Breteler MM, Ware JH, Grodstein F (2004). Physical activity, including walking, and cognitive function in older women. JAMA.

[9] Whelton SP, Chin A, Xin X, He J (2002). Effect of aerobic exercise on blood pressure: a meta-analysis of randomized, controlled trials. Ann Intern Med.

[10] Delaney EP, Greaney JL, Edwards DG, Rose WC, Fadel PJ, Farquhar WB (2010). Exaggerated sympathetic and pressor responses to handgrip exercise in older hypertensive humans: role of the muscle metaboreflex. Am J Physiol Heart Circ Physiol.

[11] Middlekauff HR, Nitzsche EU, Hoh CK, Hamilton MA, Fonarow GC, Hage A, Moriguchi JD (2000). Exaggerated renal vasoconstriction during exercise in heart failure patients. Circulation.

[12] Muller MD, Drew RC, Blaha CA, Mast JL, Cui J, Reed AB, Sinoway LI (2012). Oxidative stress contributes to the augmented exercise pressor reflex in peripheral arterial disease patients. J Physiol.

[13] Negrao CE, Rondon MU, Tinucci T, Alves MJ, Roveda F, Braga AM, Reis SF, Nastari L, Barretto AC, Krieger EM (2001). Abnormal neurovascular control during exercise is linked to heart failure severity. Am J Physiol Heart Circ Physiol.

[14] Piepoli M, Clark AL, Volterrani M, Adamopoulos S, Sleight P, Coats AJ (1996). Contribution of muscle afferents to the hemodynamic, autonomic, and ventilatory responses to exercise in patients with chronic heart failure: effects of physical training. Circulation.

[15] Fisher JP, Ogoh S, Ahmed A, Aro MR, Gute D, Fadel PJ (2007). Influence of age on cardiac baroreflex function during dynamic exercise in humans. Am J Physiol Heart Circ Physiol.

[16] Lind AR, McNicol GW (1967). Muscular factors which determine the cardiovascular responses to sustained and rhythmic exercise. Can Med Assoc J.

[17] Inder JD, Carlson DJ, Dieberg G, McFarlane JR, Hess NC, Smart NA (2016). Isometric exercise training for blood pressure management: a systematic review and meta-analysis to optimize benefit. Hypertens Res.

[18] Cornelissen VA, Smart NA (2013). Exercise training for blood pressure: a systematic review and meta-analysis. J Am Heart Assoc.

[19] Loaiza-Betancur AF, Chulvi-Medrano I (2020). Is low-intensity isometric handgrip exercise an efficient alternative in lifestyle blood pressure management? a systematic review. Sports Health.

[20] Taylor AC, McCartney N, Kamath MV, Wiley RL (2003). Isometric training lowers resting blood pressure and modulates autonomic control. Med Sci Sports Exerc.

[21] Cahu Rodrigues SL, Farah BQ, Silva G, Correia M, Pedrosa R, Vianna L, Ritti-Dias RM (2020). Vascular effects of isometric handgrip training in hypertensives. Clin Exp Hypertens.

[22] Kelley GA, Kelley KS (2010). Isometric handgrip exercise and resting blood pressure: a meta-analysis of randomized controlled trials. J Hypertens.

[23] Owen A, Wiles J, Swaine I (2010). Effect of isometric exercise on resting blood pressure: a meta-analysis. J Hum Hypertens.

[24] Yamagata T, Sako T (2020). High cardiovascular reactivity and muscle strength attenuate hypotensive effects of isometric handgrip training in young women: a randomized controlled trial. Clin Exp Hypertens.

[25] Washio T, Suzuki K, Saito S, Watanabe H, Ando S, Brothers RM, Ogoh S (2021). Effects of acute interval handgrip exercise on cognitive performance. Physiol Behav.

[26] Smolander J, Aminoff T, Korhonen I, Tervo M, Shen N, Korhonen O, Louhevaara V (1998). Heart rate and blood pressure responses to isometric exercise in young and older men. Eur J Appl Physiol Occup Physiol.

[27] Masaki M, Mitchell JH, Smith SA (2016). The exercise pressor reflex in hypertension. J Phys Fitness Sports Med.

[28] Millar PJ, MacDonald MJ, McCartney N (2011). Effects of isometric handgrip protocol on blood pressure and neurocardiac modulation. Int J Sports Med.

[29] Saito S, Washio T, Watanabe H, Tamiya K, Yamada H, Ando S, Ogoh S (2020). Effect of different interval handgrip protocol on cognitive performance. J Phys Fitness Sports Med.

[30] Sugimoto T, Suga T, Tsukamoto H, Tomoo K, Dora K, Hashimoto T, Isaka T (2020). Effect of repeated bouts versus a single bout of moderate-intensity exercise on postexercise inhibitory control. Physiol Rep.

[31] Ichinose Y, Morishita S, Suzuki R, Endo G, Tsubaki A (2020). Comparison of the effects of continuous and intermittent exercise on cerebral oxygenation and cognitive function. Adv Exp Med Biol.

[32] Tomoo K, Suga T, Sugimoto T, Tanaka D, Shimoho K, Dora K, Mok E, Matsumoto S, Tsukamoto H, Takada S (2020). Work volume is an important variable in determining the degree of inhibitory control improvements following resistance exercise. Physiol Rep.

[33] Wiley RL, Dunn CL, Cox RH, Hueppchen NA, Scott MS (1992). Isometric exercise training lowers resting blood pressure. Med Sci Sports Exerc.

[34] Akagi R, Tonotsuka M, Horie R, Hirata K, Ando S (2019). Effect of acute eye fatigue on cognition for young females: a pilot study. PeerJ.

[35] Lefferts WK, Babcock MC, Tiss MJ, Ives SJ, White CN, Brutsaert TD, Heffernan KS (2016). Effect of hypoxia on cerebrovascular and cognitive function during moderate intensity exercise. Physiol Behav.

[36] Svebak S, Murgatroyd S (1985). Metamotivational dominance: a multimethod validation of reversal theory constructs. J Pers Soc Psychol.

[37] Lakens D (2013). Calculating and reporting effect sizes to facilitate cumulative science: a practical primer for t-tests and ANOVAs. Front Psychol.

[38] Kleinloog JPD, Mensink RP, Ivanov D, Adam JJ, Uludag K, Joris PJ (2019). Aerobic exercise training improves cerebral blood flow and executive function: a randomized, controlled cross-over trial in sedentary older men. Front Aging Neurosci.

[39] Dempster K, McGowan CL, Wade TJ, O'Leary D (2018). Effects of isometric handgrip exercise training on systemic arterial stiffness, cardiovagal baroreflex sensitivity, and cognition in adults with hypertension: a pilot study. Crit Rev Phys Rehabil Med.

[40] Middleton LE, Barnes DE, Lui LY, Yaffe K (2010). Physical activity over the life course and its association with cognitive performance and impairment in old age. J Am Geriatr Soc.

[41] Erickson KI, Voss MW, Prakash RS, Basak C, Szabo A, Chaddock L, Kim JS, Heo S, Alves H, White SM (2011). Exercise training increases size of hippocampus and improves memory. Proc Natl Acad Sci USA.

[42] Hajjar I, Hart M, Mack W, Lipsitz LA (2015). Aldosterone, cognitive function, and cerebral hemodynamics in hypertension and antihypertensive therapy. Am J Hypertens.

[43] Vincent C, Hall PA (2015). Executive function in adults with type 2 diabetes: a meta-analytic review. Psychosom Med.

[44] Barnes DE, Yaffe K, Satariano WA, Tager IB (2003). A longitudinal study of cardiorespiratory fitness and cognitive function in healthy older adults. J Am Geriatr Soc.

[45] Ogoh S, Tsukamoto H, Hirasawa A, Hasegawa H, Hirose N, Hashimoto T (2014). The effect of changes in cerebral blood flow on cognitive function during exercise. Physiol Rep.

[46] Tsukamoto H, Suga T, Takenaka S, Tanaka D, Takeuchi T, Hamaoka T, Isaka T, Ogoh S, Hashimoto T (2016). Repeated high-intensity interval exercise shortens the positive effect on executive function during post-exercise recovery in healthy young males. Physiol Behav.

[47] Byun K, Hyodo K, Suwabe K, Ochi G, Sakairi Y, Kato M, Dan I, Soya H (2014). Positive effect of acute mild exercise on executive function via arousal-related prefrontal activations: an fNIRS study. Neuroimage.

[48] Tsukamoto H, Suga T, Takenaka S, Takeuchi T, Tanaka D, Hamaoka T, Hashimoto T, Isaka T (2017). An acute bout of localized resistance exercise can rapidly improve inhibitory control. PLoS ONE.

